# Antiferromagnetic Spin Coupling between Rare Earth Adatoms and Iron Islands Probed by Spin-Polarized Tunneling

**DOI:** 10.1038/srep13709

**Published:** 2015-09-03

**Authors:** David Coffey, José Luis Diez-Ferrer, David Serrate, Miguel Ciria, César de la Fuente, José Ignacio Arnaudas

**Affiliations:** 1 Laboratorio de Microscopías Avanzadas, Instituto de Nanociencia de Aragón, Universidad de Zaragoza, Zaragoza, Spain; 2 Instituto de Ciencia de Materiales de Aragón, Consejo Superior de Investigaciones Científicas, Zaragoza, Spain; 3 Departamento de Física de la Materia Condensada, Universidad de Zaragoza, Zaragoza, Spain

## Abstract

High-density magnetic storage or quantum computing could be achieved using small magnets with large magnetic anisotropy, a requirement that rare-earth iron alloys fulfill in bulk. This compelling property demands a thorough investigation of the magnetism in low dimensional rare-earth iron structures. Here, we report on the magnetic coupling between 4f single atoms and a 3d magnetic nanoisland. Thulium and lutetium adatoms deposited on iron monolayer islands pseudomorphically grown on W(110) have been investigated at low temperature with scanning tunneling microscopy and spectroscopy. The spin-polarized current indicates that both kind of adatoms have in-plane magnetic moments, which couple antiferromagnetically with their underlying iron islands. Our first-principles calculations explain the observed behavior, predicting an antiparallel coupling of the induced 5d electrons magnetic moment of the lanthanides with the 3d magnetic moment of iron, as well as their in-plane orientation, and pointing to a non-contribution of 4f electrons to the spin-polarized tunneling processes in rare earths.

The study of single magnetic atoms adsorbed on solid surfaces of different elements has been a subject of steady interest during the past two decades, mainly as concerns to adatoms of transition metals (TM). Adatoms of different 3d magnetic TM deposited on various kinds of substrates have been fairly well studied by scanning tunneling spectroscopy (STS), spin-polarized scanning tunneling microscopy (SP-STM), and inelastic scanning tunneling spectroscopy (IETS)^1^,^2^,^3^,^4^,^5^,^6^,^7^,^8^,^9^,^10^,^11^,^12^,^13^,^14^,^15^,^16^,^17^. The capability of SP-STM to reveal the spin state down to the single-atom level has led to experimental findings which encouraged further research work for a better understanding of the magnetic interactions of individual adatoms and larger nanostructures on surfaces^6^,^9^,^18^. Furthermore, looking for larger magnetic moments and magnetic anisotropy, two quantities to keep as large as possible in nanomagnetic materials designed for ultra-high density magnetic storage media and advanced spintronic devices, Rare Earth (RE) elements have to be considered. This is what has fostered the research in single ion RE molecular magnets over the last years^19^. However, for RE adatoms on surfaces, excepting the case of Ce, which attracted some early interest associated with Kondo effect^20^,^21^,^22^, only few other lanthanides: Gd^23^,^24^, Ho^25^ and Tm^26^ have been recently investigated by using local probe techniques, on occasion combined with X-ray spectroscopy experiments^26^,^27^. Nevertheless, the role of 4f electrons in the tunneling process has not been thoroughly investigated, and the accessibility of these inner electrons, recently addressed in RE molecular magnets^28^, remains an open issue for the case of RE adatoms on metals^23^,^25^.

In RE metals the 4f electrons are the main responsible for the magnetic moment of the atom and the external 5d and 6 s electrons make only a discreet contribution to it. Nevertheless, their role cannot be disregarded, since these outer electrons mediate the magnetic interactions among different atoms and greatly determine the magnetic properties of the element or compound^29^,^30^. In particular, the RE-transition metal compounds have been thoroughly studied^31^, and the magnetic coupling between the RE and TM sublattices explained through the model proposed by Campbell^32^, in which the RE-5d/TM-3d bands hybridization results in an antiparallel spin coupling, and the RE-4f and 5d spins are ferromagnetically exchange coupled^30^,^33^,^34^.

Here we report on the determination of the spin polarization state of single RE (RE = Tm and Lu) adatoms on a surface consisting of Fe monolayer (ML) nanoisland pseudomorphically grown on a W(110) surface. Our SP-STM measurements demonstrate a conduction mechanism for 4f metals involving 5d electrons and prove that these 5d-states have a net spin moment, which couples antiferromagnetically (AFM) to the overall 3d-spin moment of the Fe island, for both types of RE adatoms, independently of the existence or not of a 4f magnetic moment.

By selecting two lanthanide adatoms very close to one another, such as Tm and Lu, we deal with two adspecies of similar radii (both, the ionic^3+^ and the metallic radii, differ in less than 2 pm for the two elements) and with comparable outer electron shells; therefore, we would expect a similar chemisorption process. Nevertheless, the magnetism of these atoms is quite different, since the magnetogenic 4f shell is filled for Lu but not for Tm, which has twelve 4f electrons^26^,^29^. These 4f states are strongly localized and shielded by the 5 s and 5p electrons preventing their interaction with the surface electronic sea which, in contrast with 3d metals, precludes the quenching of the orbital moment. In STM experiments performed on 3d metals, conduction electrons from the tip preferentially tunnel into s-p-d-states of the adatom more extended into the vacuum^14^. The similar behavior that we have observed for the magnetic and non magnetic lanthanide coupled to Fe allows us to discern the nature of the tunneling electrons, pointing to the 5d as the main actor in the SP tunneling process in RE metals. Our results can shed light on a recent controversy concerning IETS experiments on different RE adatoms (Gd and Ho on Pt (111) and Cu (111)) adsorbed on conducting substrates^23^,^25^. In these experiments, the inelastic scattering processes is attributed to spin flip events of the 4f electrons with long spin lifetimes (up to 700 s at 0.7 K for Ho on Pt(111)) and, hence, with single ion magnetic anisotropy. However, in a recent paper^27^, XAS and XMCD experiments have shown that the ground state of Ho is incompatible with long spin relaxation times, which contradicts the IETS results. In this regard, the results that we report on this letter indicate a non-contribution of 4f electrons to the spin-polarized tunneling processes in RE single atoms on metals, and therefore would not expect to observe an inelastic process related to the 4f shell, suggesting that the IETS spectra might have a different origin, which supports the conclusions of the above mentioned X-ray experiments.

## Results and Discussion

### Spin-Polarized STM experiments

We studied thulium and lutetium adatoms on iron monolayer (ML) islands growth on a tungsten (110) single crystal by using spin polarized scanning tunneling microscopy. The differential conductivity (dI/dV) retrieved by a lock-in amplifier (modulating at 831 Hz, 1-10 mV rms) shows differences that can be attributed to the spin state of the sample; to investigate this, constant current and constant height dI/dV maps have been obtained (see methods and Supplementary Information^35^).

### Tm adatoms on Fe-ML/W(110)

**Figure 1:**
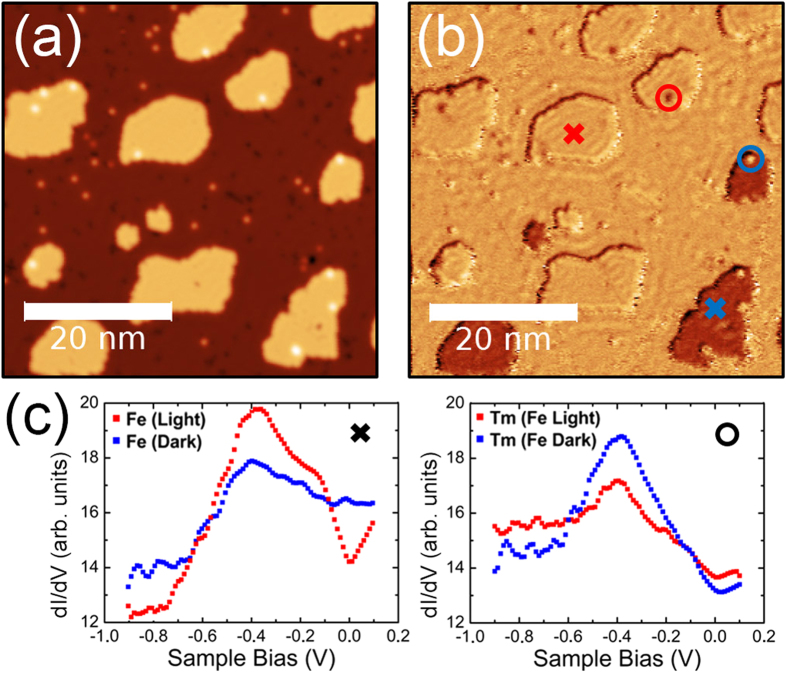
STM, SP-STM and STS of thulium adatoms and monolayer iron islands on W(110). (**a**) STM topograph of Tm adatoms adsorbed onto Fe ML nanoislands on W(110) at T = 4.5 K. Tm single atoms are seen as white spots on the Fe islands and orange ones on the bare W(110) surface (*I*_*t*_ = 1 nA, *V*_*bias*_ = −200 mV). (**b**) Simultaneously recorded spin-polarized dI/dV map of the area shown in (**a**); the dark Fe islands have in-plane magnetization contrarily aligned to the light colored ones with respect to the tip magnetization and the Tm adatoms present a contrast opposite to the one shown by the respective Fe islands beneath (*I*_*t*_ = 1 nA, *V*_*bias*_ = −200 mV; *V*_*mod*_ = 10 mV). (**c**) left: spin-resolved dI/dV spectra acquired over two Fe islands with opposite spin orientations; right: same as left but now above two Tm adatoms, sited on the Fe islands of opposite spin orientation.

**Figure 2:**
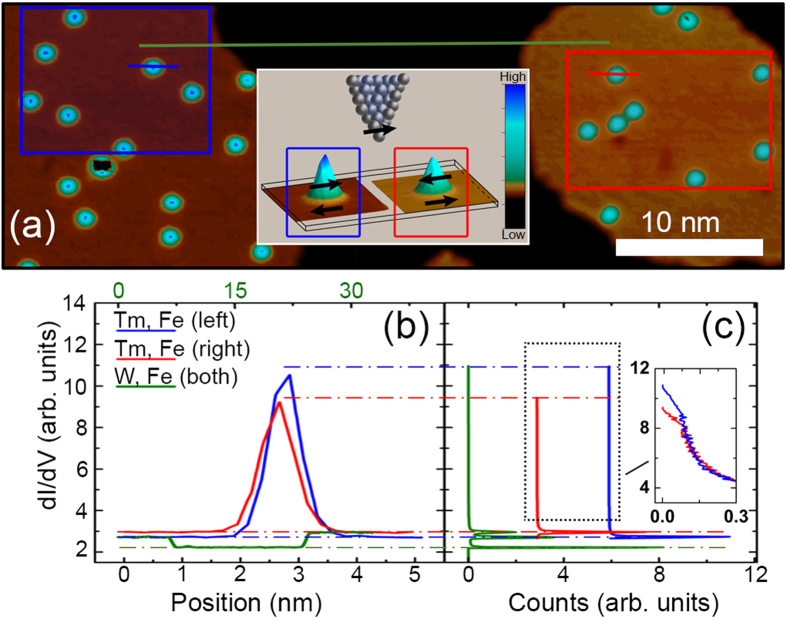
Antiferromagnetic spin coupling of thulium adatoms and iron monolayers. (**a**) Spin-polarized constant-height dI/dV map (*V*_*bias*_ = −200 mV; *V*_*mod*_ = 2 mV) of Tm adatoms adsorbed on two Fe ML islands on W(110). (**b**) Line scans through the centers of the two Tm adatoms marked by the short segments in (**a**) (*I*_*t*_ = 3 nA, *V*_*bias*_ = −900 mV; *V*_*mod*_ = 4 mV). (**c**) Histograms of the dI/dV values for the whole area displayed in (**a**), green line histogram, and for the two regions delimited by red and blue rectangles (island and tip magnetization parallel and antiparallel, respectively), red and blue lines. Data sets are offseted in the horizontal axis for clarity. Inset in (**a**): visualization of the proposed spin configurations. Inset in (**c**) shows in detail the dI/dV distribution near the adatom apices, with no horizontal offset for better comparison.

Figure 1(a) shows a constant current STM topography of a large area in which Fe monolayer islands (light orange) of average size of about 10 nm are distributed. The Tm adatoms appear as light-orange spots on top of the W(110) substrate and as white ones over the Fe islands. In Fig. 1(b) the spin-polarized local conductance map of the same area at −200 mV displays clearly two kinds of Fe islands. Since the Fe monolayers grown on W(110) have in-plane magnetization, with easy magnetization axis along [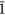
10]^36^,^37^, the observed contrast indicates that the dominant tip magnetization component is in plane, either parallel or antiparallel to the magnetic moment of the islands underneath. This is corroborated by the dI/dV spectra shown in Fig. 1(c left) performed on two Fe islands displaying different contrast. It is clear that 200 meV below the Fermi energy [the dI/dV map in Fig. 1(b)], the conductance for both islands is different. More interesting is the behavior of the Tm adatoms, which display a contrast opposite to the one shown by the respective Fe islands beneath, as seen on the dI/dV map of Fig. 1(b) and further detailed by the spectra of Fig. 1(c, right) performed on two Tm adatoms on islands of opposite contrast. This result suggests that the adatoms bear a net spin moment that is antiferromagnetically coupled to the magnetic moment of the underlying Fe island. However, a possible dependence of the adatom’s spin polarization on the energy and vertical distance to the tip, certainly found in Co and Cr atoms on Fe islands on W(110)^14^, should be analyzed in our case. To discard an artifact associated with changes in the tunneling current because of the tip height, related with the different spatial decays of the different orbital characters of majority and minority states^14^, we performed SP-dI/dV maps and spectra in the constant-height operation mode. Two close generic Fe islands with several Tm adatoms on them are displayed in Fig. 2(a), this dI/dV map was performed after positioning the tip over a Tm adatom on an island at *V*_*bias*_ = −200 mV, *I*_*t*_ = 800 pA and opening the feedback loop. The profile shown in Fig. 2(b) clearly reveals that the right island (baseline of the red peak), has a larger dI/dV value than the left island (baseline of the blue peak) and that the Tm adatom on top of the right island has a lower dI/dV signal than the one on top of the left Fe island. This implies that the spin polarization of islands and adatoms are opposite to each other. The same holds true for the range between −900 and +100 mV [see Fig. 1(c)], which can only be rationalized if the magnetization of adatoms and islands are antiparallel in a large energy range around the Fermi level.

We find the same behavior in all Tm adatoms on islands with opposite spin contrast and with several SP tips. As a more representative dI/dV signal analysis of a particular region, we have performed histograms as those shown in Fig. 2(c). The green histogram corresponds to the whole area: the minimal conductance has the maximum number of events, i. e., the W(110) free surface area produces the larger green peak; the Fe island with magnetization antiparallel to the tip moment produces the second higher green peak and, close to it and slightly lower but at a higher value of dI/dV, the third peak comes from the Fe island parallel to the tip magnetization. The two other histograms, blue and red lines, are horizontally shifted for clarity and correspond to the two regions delimited by blue and red rectangles, respectively, in Fig. 2(a). These regions have no contribution from the substrate, so that the largest peak at the lower value of dI/dV is missing, and allow a clearer comparison of the conductance of each iron island with the adatoms on them. The island with a lower conductance (peak in the blue histogram) reaches the maximum values of conductance for a few events, the areas occupied by the *cusps* of the Tm adatoms. The opposite happens for the other Fe island, it has a higher conductance (peak in the red histogram) but the adatoms on it do not reach such large dI/dV values as their counterparts on the other island.

### Lu adatoms on Fe-ML/W(110)

**Figure 3:**
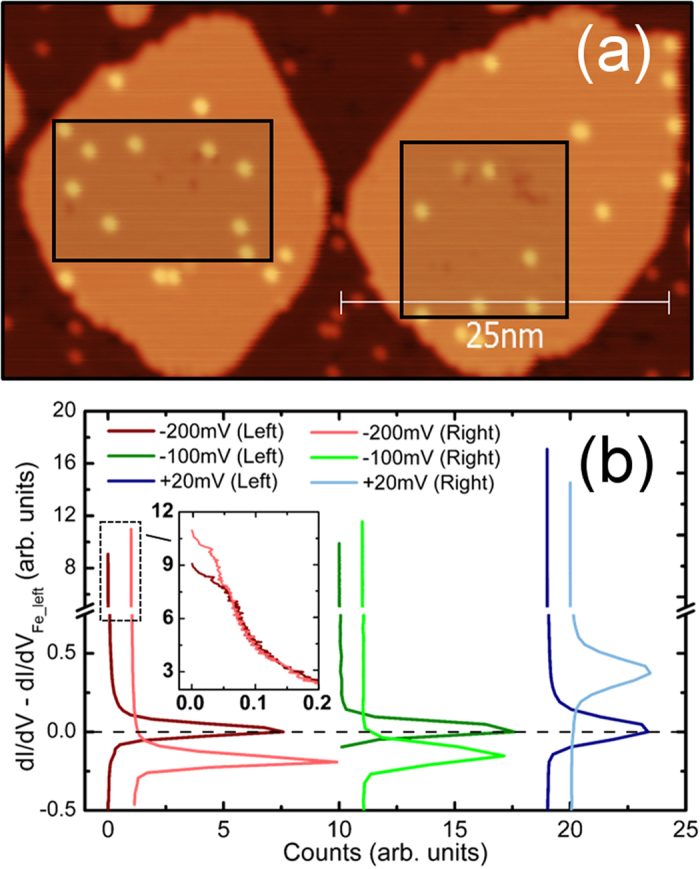
Spin-polarized conductance of lutetium adatoms on iron monolayer islands. (**a**) Constant current topography map of the area of interest. (**b**) Histograms of constant-height dI/dV values for areas containing two oppositely polarized Fe ML islands and Lu adatoms. Red pair of histograms corresponds to *V*_*bias*_ = −200 mV, green to *V*_*bias*_ = −100 mV and blue to *V*_*bias*_ = +20 mV. For easier comparison, each pair of histograms has been vertically shifted so that the signal from Fe island on the left is zero in all three cases. Note the simultaneous change of polarization of both, the islands and their Lu adatoms, depending on the bias voltage. At −200 mV and −100 mV the island on the left is parallel to the tip moment and the Lu adatoms on it are antiparallel; for +20 mV the situation is reversed, it is the island on the right that is parallel to the tip and its Lu adatoms, antiparallel. Inset in (**b**) shows in detail the dI/dV distribution near the adatom apices at −200 mV, with no horizontal offset for better comparison.

The SP-STM constant current measurements on the samples with Lu adatoms provide similar images to the shown for Tm in Fig. 1(b). Moreover, an analysis as done for Tm of constant-height spin-resolved conductance maps (see Fig. 3), indicates that the Lu adatoms, which as free atoms do not possess magnetic moment, display a spin polarization also opposite to that of the Fe ML island on which they are adsorbed. The antiferromagnetic coupling is proved, not only because for a given energy range the measurements are performed at constant-height and we are measuring the tunneling current at the same vacuum region, but also because we observe a simultaneous reversal of the contrast when going to different energies in the dI/dV spectra (compare Fig. 3: −200 mV and −100 mV to +20 mV and, for more details, see Supplementary Information^35^). Our results for Lu suggested us to ascribe its detected spin moment, as well as that for Tm, not to the 4f electrons but to the outer shells electrons, which are prone to hybridize with the 3d electrons of Fe and acquire a net spin moment.

### First-principles calculations

To support the above interpretation of our experimental results we have investigated the electronic and magnetic properties of Tm and Lu adatoms on a Fe monolayer on W(110) by using DFT-based first-principles calculations within the generalized gradient approximation using the projector-augmented wave method^38^ (see methods). In Table 1 we summarize the most relevant data obtained regarding the experiments: the orbital decomposition of the spin magnetic moment at the Fe, Tm and Lu sites, along the [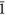
10] direction. The spin moments are practically aligned along it, the magnetic easy direction of the Fe monolayer (components along other directions are found negligible, except perhaps a 4f spin moment of +0.14 *μ*_*B*_ obtained for the Tm adatom along the [110] direction). The significant result in our case is that the 5d electrons for both, Tm and Lu adatoms, display a net spin moment and that it has the opposite direction to the 3d spin moment of Fe. Moreover, since, in the case of Tm, the obtained 4f spin moment is FM coupled with the 5d spin moment, the total spin moment of this RE is AFM coupled to the Fe ML. For Lu, as expected, the spin moment arises mainly from the 5d electrons and couples AFM with the 3d spin moment of the Fe island. It is the adsorption of the RE adatom on the transition metal which, by hybridization with the 3d bands, allows the 5d orbitals of the RE or to be populated in the case of Tm (as free atom Tm has no 5d electrons), or more occupied for Lu (with one 5d electron as free atom). Since these 5d orbitals are polarized, we get a net 5d spin moment in both cases and, because those orbitals are much more external than the 4f ones, the tunneling current becomes spin-polarized, as seen in the present experiments, being such spin polarization opposite to that of the Fe ML on which the adatom is adsorbed.

On the other hand, the calculated RE 4f to 5d DOS ratio near the Fermi level is ~8 × 10^−4^, implying that, at the energies involved in the SP-STM measurements, the main contribution to the tunneling current from the RE adatoms comes from the 5d electrons.

## Conclusion

In conclusion, by using spin-polarized scanning tunneling microscopy and spectroscopy in combination with first-principles calculations, we report on the magnetic coupling existing between Tm and Lu adatoms and Fe monolayer islands with in-plane magnetization: the RE 5d moments lie in plane and couple antiferromagnetically with their underlying iron islands. This case represents an extreme manifestation of the Campbell mechanism^32^, till now only tested in higher dimensionality structures and bulk RE-TM alloys. Furthermore, we show that the RE-TM interaction proceeds via the 3d–5d direct AF interaction, that 4f–5d intra atomic FM exchange also holds at the single atom limit and that the RE 4f electrons have a negligible contribution to the tunneling current.

## Methods

### Samples preparation and experimental details

The W(110) crystal was prepared by successive cycles of annealing at 1500 K in an oxygen atmosphere (p < 5 × 10^−8^ mbar) and flashing at 2300 K in ultrahigh vacuum conditions (p < 2 × 10^−10^ mbar). Prior to the Fe evaporation, the absence of surface contamination was confirmed by low temperature STM, followed by another flash to ensure a clean surface. 0.3 ML of Fe were evaporated with the sample at approximately *T*_*sample*_ ≈ 550 K, obtaining large (≈30 nm) single layer Fe islands. Tm and Lu atoms were deposited on the Fe/W sample directly on the STM stage, at 5 K, by electron beam evaporation. In-plane spin polarized STM tips were prepared from electro-chemically etched tungsten wire, flashed in UHV conditions and coated with 90 ML of Fe. All measurements were performed in UHV conditions using a SPECS JT-STM at 4.2 K.

### First-principles calculations

The electronic and magnetic properties of Tm and Lu adatoms on a Fe monolayer on W(110) have been investigated by using DFT-based first-principles calculations with the generalized gradient approximation (GGA) for correlation and exchange in the used local spin density approximation (LSDA) and the plane basis is set on projector augmented wave (PAW) pseudopotential for describing the core electrons as implemented in Vienna ab-initio simulation package (VASP)^38^. As is well established, the LSDA often fails to describe systems with localized strong correlated d and f electrons, which can be overcome by introducing LSDA + U method (we used its rotationally invariant generalization)^39^. Finally, the spin-orbit coupling (SOC) (using the fully unconstrained noncollinear method)^40^ was included to study the dependence of Khon-Sham ground state energy on the direction of the magnetic moment. Full electronic relaxation was carried out for a lanthanide adatom (Tm or Lu) on a ferromagnetic ML of Fe over a 5-ML-thick W(110) slab, using (3 × 3 × 1) supercells and a 3 × 3 × 1 k-mesh Γ-centered. More details of the procedure followed in the calculations and complete results can be found in the Supplementary Information^35^.

## Additional Information

**How to cite this article**: Coffey, D. *et al*. Antiferromagnetic Spin Coupling between Rare Earth Adatoms and Iron Islands Probed by Spin-Polarized Tunneling. *Sci. Rep*. **5**, 13709; doi: 10.1038/srep13709 (2015).

## Supplementary Material

Supplementary Information

## Figures and Tables

**Table 1:** Spin magnetic moments (in *μ*_*B*_/atom) along [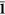
10] at the Fe, Tm and Lu sites, decomposed by the s, p, d, f-character of the electrons.

	s	p	d	f	total
Tm/Fe/W(110)					
Tm	−0.02	0.00	−0.13	−0.92	−1.07
Fe	0.01	−0.01	2.45	—	2.45
Lu/Fe/W(110)					
Lu	−0.02	0.00	−0.17	0.02	−0.17
Fe	0.02	−0.01	2.34	—	2.36
