# Supplementary material for: Antiferromagnetic Spin Coupling between Rare Earth Adatoms and Iron Islands Probed by Spin-Polarized Tunneling
Source: Sci Rep. 2015 Sep 3;5:13709. doi: 10.1038/srep13709 (PMC4558548; doi:10.1038/srep13709)
Supplement: Supplementary Information [file srep13709-s1.pdf]

Supplementary information

Antiferromagnetic Spin Coupling between Rare Earth Adatoms and Iron Islands Probed by Spin-Polarized Tunneling

David Coffey,[*,+,§,#] José Luis Diez-Ferrer,[+] David Serrate,[+,#] Miguel Ciria,[§,#] César de la Fuente [§,#] and José Ignacio Arnaudas [*,+,#]

Laboratorio de Microscopías Avanzadas, Instituto de Nanociencia de Aragón, Universidad de Zaragoza, 50018 Zaragoza, Spain, and Instituto de Ciencia de Materiales de Aragón (ICMA), Consejo Superior de Investigaciones Científicas (CSIC) - Universidad de Zaragoza, 50009 Zaragoza, Spain, and Departamento de Física de la Materia Condensada, Universidad de Zaragoza, 50071 Zaragoza, Spain

E-mail: dcoffeyb@unizar.es; arnaudas@unizar.es

* To whom correspondence should be addressed

\+ Laboratorio de Microscopías Avanzadas, Instituto de Nanociencia de Aragón, Universidad de Zaragoza, c/Mariano Esquillor, s/n, 50018-Zaragoza, Spain

§ Instituto de Ciencia de Materiales de Aragón (ICMA), CSIC-Universidad de Zaragoza, c/Pedro Cerbuna, 12, 50009-Zaragoza, Spain

Departamento de Física de la Materia Condensada, Universidad de Zaragoza, 50071-Zaragoza, Spain

SUPPLEMENTARY STM MEASUREMENTS ON RE/Fe/W(110)

The differential conductivity (dI/dV) retrieved by a lock-in amplifier portrays the spatially resolved spin state of the sample, as the dI/dV signal has a component proportional to P1·P2·cos(θ),[1] where P1 (P2) is the spin polarization of the sample (tip) and θ the angle formed by the tip magnetization and the local magnetic moment of the sample. The sign of P1 and P2 determines whether parallel (θ = 0º) or antiparallel (θ = 180º) alignment provides the highest tunneling conductance state. Without loss of generality, here we assume that P of the Fe-coated W tip and the Fe ML on W(110) have positive sign.

In our study, we have focused our attention on dI/dV maps taken with an open feedback, to ensure that the tip-sample distance is the same for islands of either polarization, and for atoms on either island. Measurements taken using the usual constant current mode can be distorted when using a spin-polarized tip, as different magnetic configurations can give different contributions to I_t so the tip-sample distance is changed to maintain a constant current. The dI/dV signal is affected by this, complicating its interpretation; to avoid this problem we have taken our dI/dV maps using an open feedback: after correcting the scan angle to account for the sample tilt and letting the tip in tunnel for a long time (≥ 24 hours), scanning the area of interest until no drift is observed, the tip is set on top of the highest feature, an atom on an island, and a tunnel current of the order of 800 pA is set after which the feedback is released and the tip scans in a plane parallel to the sample. With this setup, we ensure that the tip-atom and tip-island distances remain the same regardless of the spin-polarized contribution.

The following images show how crucial this effect can be, and how a constant current dI/dV map could lead to an erroneous conclusion. Fig. S1(a) shows a constant current topography map of an area with three monolayer Fe islands on W(110) which we would expect to present the same height if measured with a non-SP tip, however if we take a profile (Fig. S1(b)) on this image we can observe that the island on the right appears to be slightly larger (16 pm) than the other two, due to having a different magnetic orientation, which means that to keep a constant current the tip-sample distance is actually larger on this island than on the other two. Fig. S1(c) shows a constant current

dI/dV map of these islands, while Fig. S1(d) shows an open-feedback dI/dV map of the same area, at the same bias and using the same modulation as Fig. S1(c).

The observed contrast reversal is due to the effect of the tip-sample difference between islands being large enough to overcome the actual dI/dV signal difference between the islands, although it should be noted that at this sample bias, V_{bias} = -25 mV, the spin polarized contrast is very low and we have not observed such reversal of the island contrast at other biases (such as -200 mV) where the dI/dV difference is substantially larger.

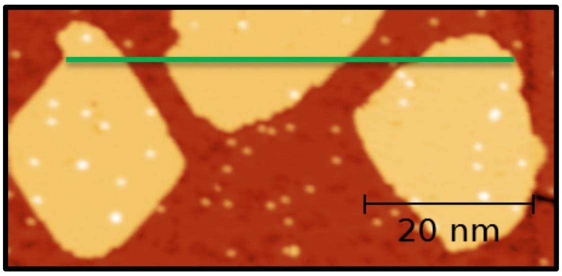

a) Topography, I_t = 300 pA, V_{bias} = - 25 mV

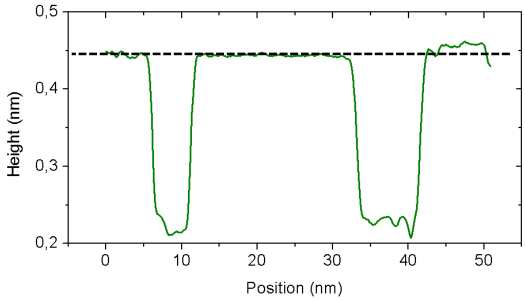

b) Profile along the green line in a)

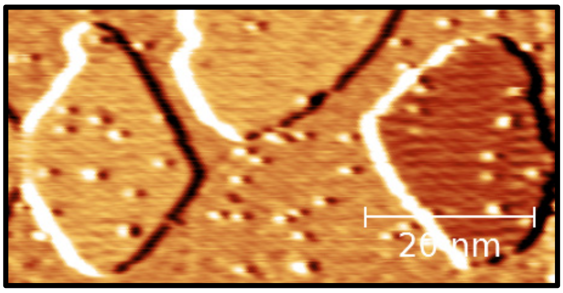

c) dI/dV map, with I_t = 300 pA, V_{bias} = - 25 mV, V_{mod} = 2 mV

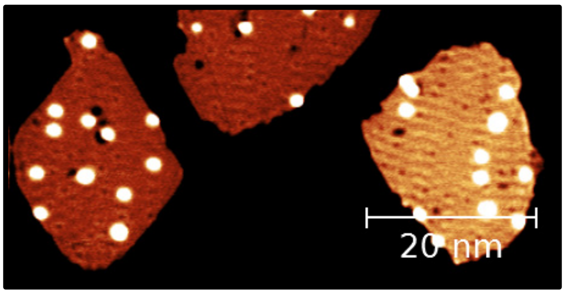

d) dI/dV map in open feedback, with V_{bias} = -25 mV, V_{mod} = 2 mV

Figure S1.- Spin-polarized-tip STM measurements at constant current (a-c) and in open feedback mode (d).

The following figure (Fig. S2) shows the open feedback dI/dV map used for Fig. 2 in main text (V_{bias} = -200 mV) as well as maps taken on different islands, at different bias (+35 mV, -35 mV). In all three cases an opposite contrast between Tm adatoms and Fe islands is observed, the dI/dV signal for adatoms on the island with lower dI/dV signal is larger than for adatoms on the island with higher dI/dV signal.

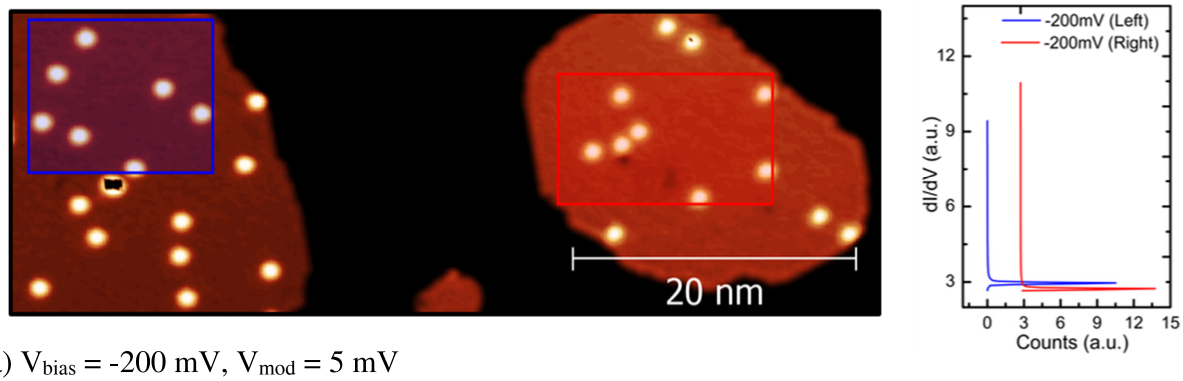

a) V_{bias} = -200 mV, V_{mod} = 5 mV

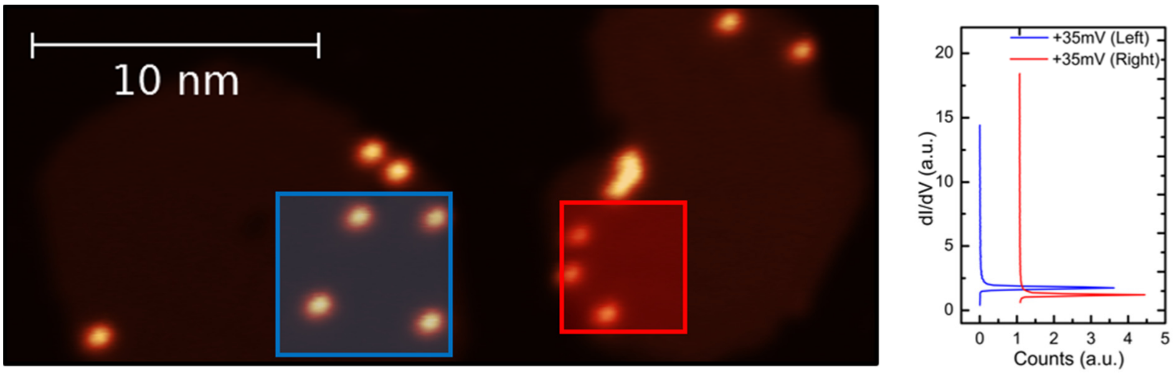

b) V_{bias} = +35 mV, V_{mod} = 1 mV

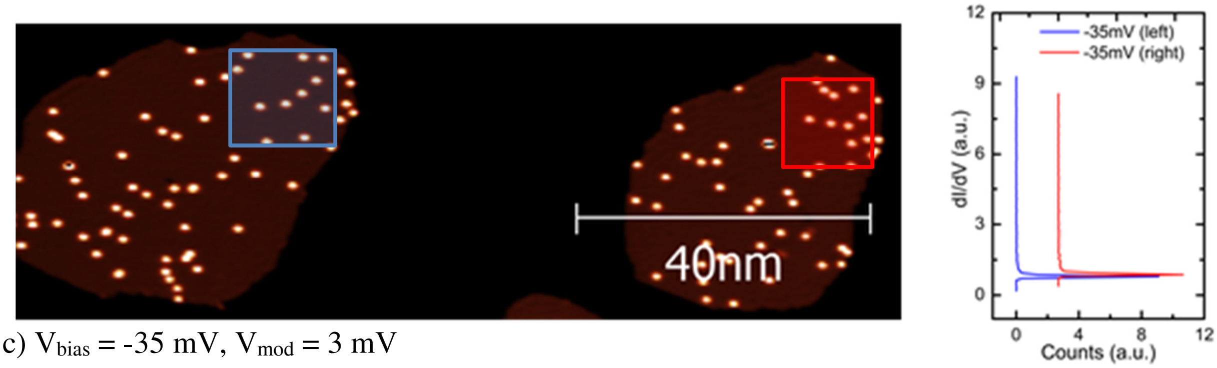

c) V_{bias} = -35 mV, V_{mod} = 3 mV

Figure S2.- Open feedback dI/dV maps used for Figure 2 in main text, for Tm/Fe/W(110).

As for Tm, an analysis of the dI/dV signal at different bias for Lu adatoms on monolayer Fe islands on W(110) shows an opposite contrast between the Lu adatoms and the Fe islands. Here we show (Fig S3) the data used for Fig. 3 in main text, studying the same set of adatoms at -200 mV, -100 mV and +20 mV sample bias. Note that the contrast inverts for the islands at +20 mV respect to -200 mV and -100 mV, as does the contrast for the Lu adatoms.

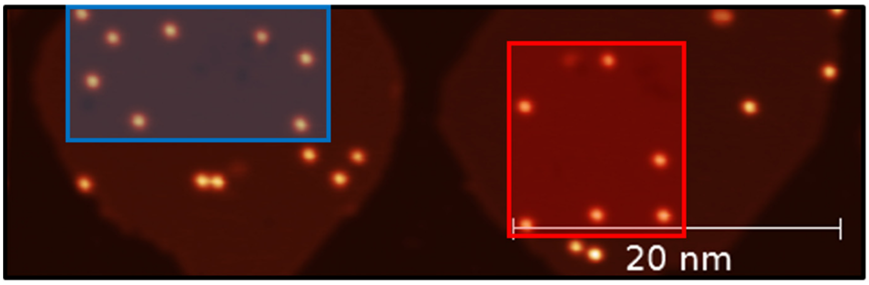

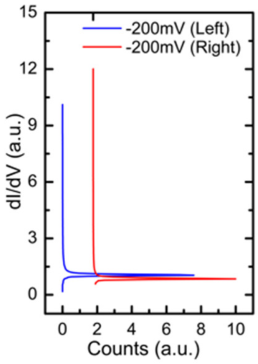

a) V_{bias} = -200 mV, V_{mod} = 5 mV

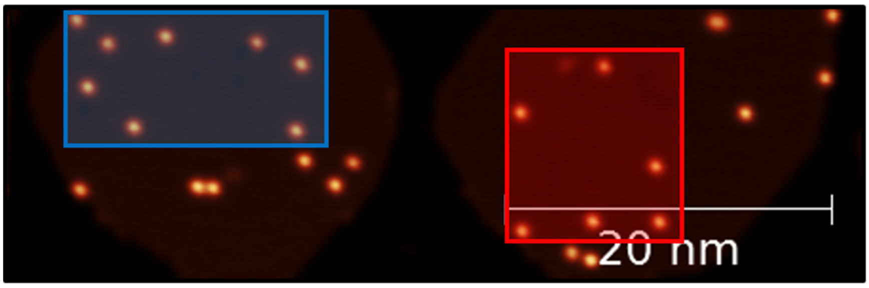

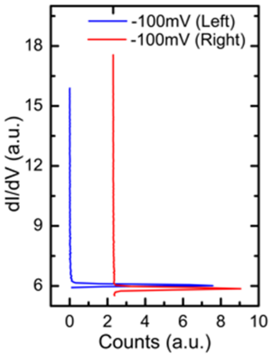

b) Lu/Fe/W(110), V_{bias} = -100 mV, V_{mod} = 5 mV

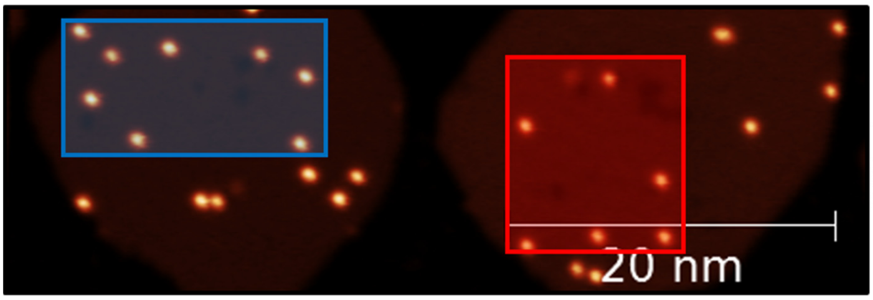

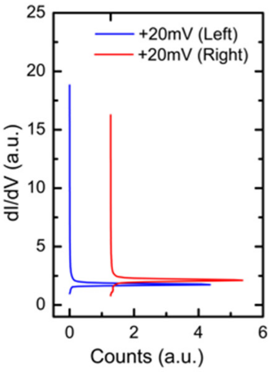

c) Lu/Fe/W(110), V_{bias} = +20 mV, V_{mod} = 0.4 mV

Figure S3.- Open feedback dI/dV maps used for Figure 3 in main text, for Lu/Fe/W(110).

AB-INITIO CALCULATIONS ON RE/Fe/W(110)

We performed a study of the electronic properties by using DFT-based first-principles calculations as implemented in Vienna ab initio simulation package (VASP).[2] We use the generalized gradient approximation (GGA) and local spin density approximation (LSDA) for correlation and exchange.[3] The pseudo-potentials method[4] allows the efficient use of plane basis set[5] with significantly fewer nodes in the description of pseudo-wave functions of core electrons. Our systems contain localized strong correlated *d* and *f* electrons, so we use the rotationally invariant LSDA+U introduced by Liechtenstein et al.[6] for including the corresponding corrections when GGA fails. Finally, the spin-orbit coupling (SOC) was also included to analyze the dependence of Khon-Sham ground state energy on the direction of the magnetic moments. In this case, we use the unconstrained “non collinear” method in VASP.[7] All calculation were done at the cluster “Memento” of Zaragoza’s University, within the “Spanish National Centre of Supercomputation”.

A) STRUCTURAL MODEL.

To obtain structural models as close as possible for our systems, we previously reproduce some of those results existing in the literature for an iron monolayer on a (110)-cut tungsten substrate. The unit cell of our final super-cell structure can be found elsewhere in the literature, for example in refs. [8–11] and refs. therein. After that, the lanthanide adatoms are included for getting final optimized structures that allow a full electronic calculation.

- <u>Fe monolayer on (110)W</u>.

The initial unit cell contains 1 ML pseudomorphic of Fe (4 atoms) over 5 ML of W oriented along [110] direction (4 atoms/layer) and a vacuum thickness of about 20 Å along [110].[8–11] Then, a (3 × 3 × 1) super-cell was built and the new structure was optimized structurally, along [110] direction. In this process, we use [Xe $4f^{14}$] $6s^2$ $5d^4$ and [Ar] $3d^7$ $4s^1$ as initial electronic configurations for W and Fe, respectively; one k-point at Γ (k = 0) and an energy cutoff of 270 eV. Initially our calculated W-bulk lattice parameter was 3.20 Å, which is larger than the experimental; but it is known that GGA overestimates bulk lattice parameters for heavy atoms. Dipolar corrections are also included by using the methods described in refs. [12,13]. During the structural optimization, we initially used a conjugate-gradient method[14] for obtaining coarse ionic positions based into their instantaneous ground state energy (after an electronic relaxation). After that, the

ionic relaxation was refined by using a quasi-Newton method[15] getting total Hellmann-Feynman forces of less than 1 mRy/a.u per atom. Let us label as "Fe / W_1 / W_2 / W_3 / W_2 / W_1" the layers of the super-cell structure. So, d(Fe-W_1) = 1.93 Å, d(W_2-W_1) = 2.25Å and d(W_3-W_2) = 2.23 Å are the distances between nearest layers, close values to those existing in the literature for a smaller unit cell.[8–11]

- Tm and Lu adatoms on Fe-ML/ (110) W super-cell

Under the above commented structure, the lanthanide adatoms, Tm and Lu, are included for the structural optimization. In this calculation, we use [Kr $4d^{10}$] $5s^2$ $5p^6$ $4f^{12}$ $6s^2$ $5d^1$ and [Kr $4d^{10}$] $5s^2$ $5p^6$ $4f^{14}$ $6s^2$ $5d^1$, as the initial electronic configuration for Tm and Lu, respectively. The initial seed position of adatoms was set at hollow site on the Fe-ML. Then, we did a fully 3D optimization for adatoms and the first two layers of the super-cell. After that, the hollow site is still the preferred equilibrium position by Tm and Lu adatoms. The optimized average distances along [110] direction are given by, d(Tm-Fe) = 2.02 Å, d(Fe-W_1) = 1.92 Å, d(W_2-W_1) = 2.24 Å and d(W_3-W_2) = 2.23 Å for Tm/Fe-ML/(110)W, and d(Lu-Fe) = 1.55 Å, d(Fe-W_1) = 1.817 Å, d(W_2-W_1) = 2.25Å and d(W_3-W_2) = 2.23 Å for Lu/Fe-ML/(110)W. No significant changes are obtained for the relative positions of the Fe and W planes, compared with Fe-ML/(110)W-case. The Fe atoms closest to Tm adatoms are locally strained, between +1.3% and –0.4%, mainly along the [110] direction, when are compared with the rest of Fe atoms belonging to the same ML. It does not happen for Lu case.

B) COLLINEAR ELECTRONIC RELAXATION.

- Fe monolayer on (110)W.

A collinear spin-polarized (SP) electronic relaxation was done by using a k-mesh of (3 × 3 × 1) centered at Γ− point. Figure S4(a) shows the total DOS for Fe monolayer for spin up and down, and we get a homogeneous ferromagnetic (FM) arrangement with a magnetic moment of +2.5 μ_B per Fe atom. This represents an increment of about +12.3% as compared with the value for bulk Fe (2.2 μ_B). The *d*-states hybridization between nearest Fe and W atoms can explain this increment. In fact, the W1-layer has a tiny magnetic moment per W atom of about 0.09 μ_B and antiferromagnetically (AF) coupled with Fe-ML. The rest of W atoms have a null magnetic moment. The analysis of 3*d*-PDOS for Fe atoms shows that the main contributions come from d_{xz} and d_{yz} states.

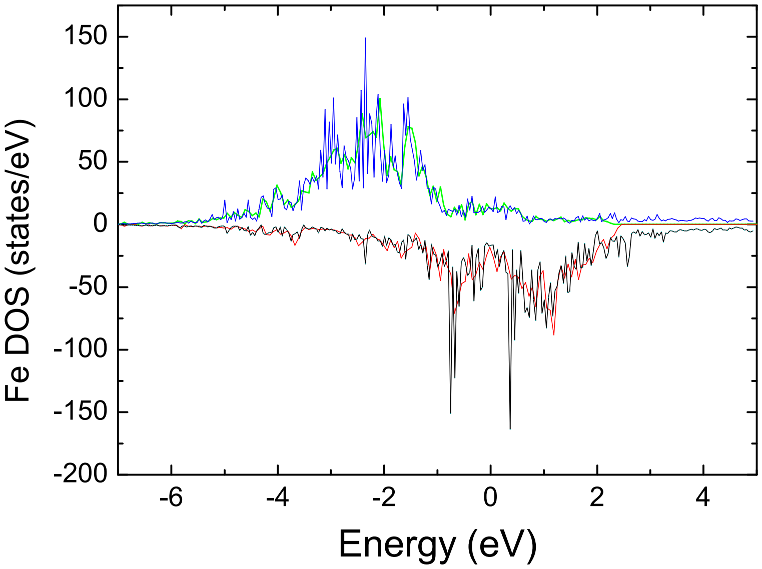

Figure S4.- The spin resolved total DOS for Fe: (a) in a Fe-ML/(110)W for (–) spin-up and (–) spin-down, and (b) in Tm/Fe-ML/(110)W for (–) spin-up and (–) spin-down. (Fermi level at 0 eV in both cases).

- Tm and Lu ad-atom on 1ML Fe/ (110)W supercell

Figure S4(b) shows the total DOS of Fe atoms for Tm/Fe-ML/(110)W, obtained from a static SP collinear electronic relaxation. The differences are small when compared with Fe-ML/(110)W case. Let us mention the existence of some resonances, mainly coming from d_{xz} and d_{yz} states, at –2.97 eV and –2.33 eV for spin-up states, and at –0.73 eV and 0.39 eV for spin-down ones. The structural optimization with Tm adatom can open new hybridization energy channels with nearest Fe atoms. Most of Fe atoms have a magnetic moment of about +2.4 μ_B, still +7.7% above the Fe bulk value. However, those Fe atoms close to the Tm adatom have a magnetic moment reduced by almost a 14%, compared with bulk value.

Figure S5 shows the 5*d*-PDOS of Tm adatom. Above the Fermi energy, 0 eV, it shows strong d_{xz} and d_{yz} contributions between 2.0 and 3.0 eV for both, minority and majority states. However, below the Fermi level the 5*d*-PDOS are small and noisy, but still dominated by d_{xz} and d_{yz}. The most noticeable values, mainly coming from minority states, appear around -450 meV, as shown in the inset, and give the major contribution to the 5*d*-magnetic moment in Tm, which amounts -0.09 μ_B. Later, we shall comment how these states could be the main responsible of experimental STM results at small but negative bias voltages between tip and sample.

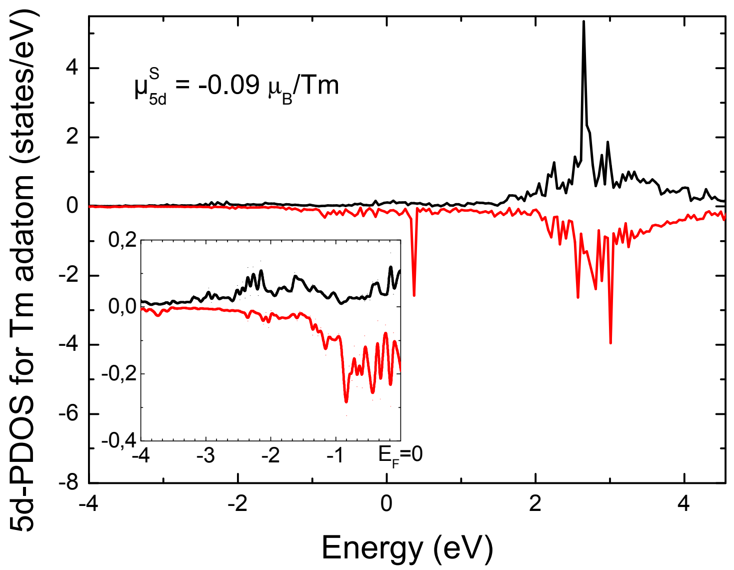

Figure S5.- The spin resolved 5*d*-PDOS of Tm adatom at Tm/Fe-ML/(110)W for (–) spin-up and (–) spin-down.

Figure S6 shows the 4*f* states obtained from calculation without U-correction. The 4*f*-spin magnetic moment of the Tm adatom is -0.96 μ_B, and is FM coupled with the corresponding 5*d* moment and, hence, AFM coupled with the rest of Fe magnetic moments. Notice that these calculations show a maximum of 4*f*-PDOS at Fermi level. These would suggest that Tm adatom behaves as a Kondo-impurity. However, our STM experimental results do not show any Kondo fingerprint at Tm impurity. Therefore, LSDA fails as expected and the energy of 4*f* states will be definitively corrected by including the U-Coulomb repulsion, when later we include SOC in the non-collinear calculations.

Finally, let us mention some results for the Lu case. There exists a close parallelism with the Tm/Fe-ML/(110)W case. All Fe magnetic moments have about +2.3 μ_B, even those Fe atoms near to the Lu adatom. Lu adatom does not show any 4*f*-spin moment because its 4*f* band is full. The 5*d*-states have a small magnetic moment of -0.1 μ_B, as Tm, and it is AFM coupled with all the rest of Fe magnetic moments, as it happened in Tm case. At this stage, collinear calculations explain our experimental SP-STM results as well as the applicability of the "Campbell mechanism"[16] at the level of the single lanthanide adatoms existing in our systems. Next, we investigate whether or not this mechanism is similarly predicted when including SOC + U in a non-collinear calculation.

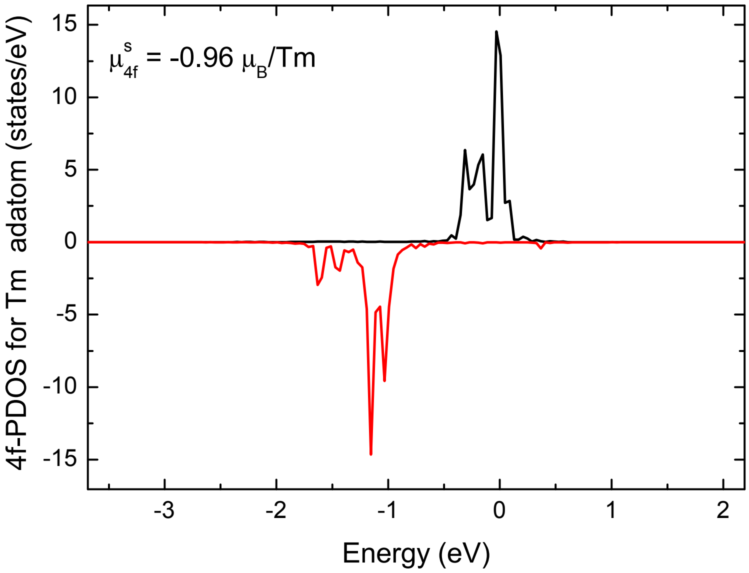

Figure S6.- The spin resolved 4*f*-PDOS of Tm adatom at Tm/Fe-ML/(110)W for (−) spin-up and (−) spin-down.

C) NON-COLLINEAR ELECTRONIC RELAXATION

- Fe monolayer on (110)W.

A non-collinear SP electronic relaxation including SOC + U was done, using [110], [$\bar{1}$10] and [001] directions as quantization axes for spin. We use U = 0.8 eV for 3*d* Fe states.[17] The spin resolved Fe PDOS along [$\bar{1}$10] is not quite different from that shown in Figure S4(a), just slightly shifted by the U correction. In all studied quantization axes, the magnetic moment for Fe atoms is about 2.4 μ_B. The final electronic structure of Fe ions is quite homogeneous within the Fe monolayer and gives: [Ar $3s^2$] $3d^6$ $4s^{0.42}$ $4p^{0.35}$.

The total energy difference between calculations with quantization axes along [001] and [$\bar{1}$10] is almost 1 meV, and it duplicates the value when this difference is between [110] and [$\bar{1}$10] directions. So, [$\bar{1}$10]-direction is the easy axis in the super-cell of Fe-ML/ (110)W, as other authors had previously found using a smaller unit cell.[11]

- Tm and Lu adatoms on 1ML Fe/ (110)W supercell

A non-collinear SP electronic relaxation including SOC + U was done by using U = 0.8 eV for 3*d*-Fe states and U = 5.0 eV for the 4*f*-adatom states.[18] The quantization axis for spin was set along the easy axis of Fe-ML, and the Fe spin resolved PDOS have no appreciable differences with respect collinear calculation, even for those Fe atoms close to Tm and Lu adatoms. Now, the 4*f*-PDOS are shifted away from the Fermi level because the U repulsion, and 5*d*-states distribution is similar to that found for the collinear calculation. Thus, the final electronic structure of Tm adatom is [Kr $4d^{10}$] f^{12} $s^{2.33}$ p^6 $d^{0.56}$ and for Lu adatom, [Kr $4d^{10}$] f^{14} $s^{2.46}$ p^6 $d^{1.22}$.

The calculated 4*f* spin moment is negligible for the Lu adatom, as expected. The Tm 4*f* spin moment has a small 8.6° tilt out from the axis $[\bar{1}10]$ toward [110] direction, within the (001) plane, having its largest component, -0.92 μ_B, along the $[\bar{1}10]$ direction. That tiny perpendicular component of the spin moment can be a consequence of the unidirectional symmetry observed by the Tm ion on the Fe island. So, the 4*f* spin of Tm adatom is nearly parallel to its 5*d* spin moment (in the case of Rare Earth-transition metal intermetallics both moments are FM coupled[16,18]). However, the Tm 5*d* and Fe 3*d* spin magnetic moments are completely aligned along the easy-axis of Fe monolayer, and are antiparallel. The 5*d*-spin magnetic moments have similar values to those found in the collinear calculations, -0.13 μ_B for Tm case and -0.17 μ_B for Lu. The Campbell mechanism still holds taking into account SOC+U corrections.

Now that we have a full non-collinear ab-initio calculation with SOC+U, we would like to correlate some of the experimental results obtained by STM with ab-initio results. Therefore, we calculate the spin-averaged conductance, dI/dV, at different bias between tip and our samples. Figure S7 shows the spin-averaged conductance for Tm/Fe-ML/(110)W, the conductance above the Tm adatom shows an important contribution at high and positive bias and the agreement between calculation and experimental result is fine. An analysis of Tm 5*d*-PDOS (see Figure S8) allows to see the different contributions to the conductance coming from the five *d*-orbitals. It is worth noting that the calculated 4*f* to 5*d* DOS ratio, between -1 eV and 140 meV, is ~ $8\text{x}10^{-4}$ and barely $7\text{x}10^{-3}$ at higher energies, up to 2.7 eV. Therefore, at the energies involved in the SP-STM measurements, the main contribution to the tunneling current when the tip is over Tm or Lu adatoms comes from the RE 5*d*-electrons.

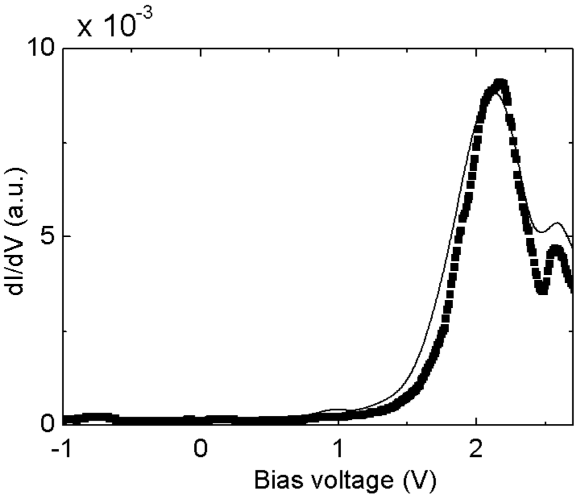

Figure S7. Experimental spin-averaged conductance for Tm/Fe-ML/(110)W, with the tip at 3.0 Å over the Tm adatom (■). The continuous line represents the calculated conductance.

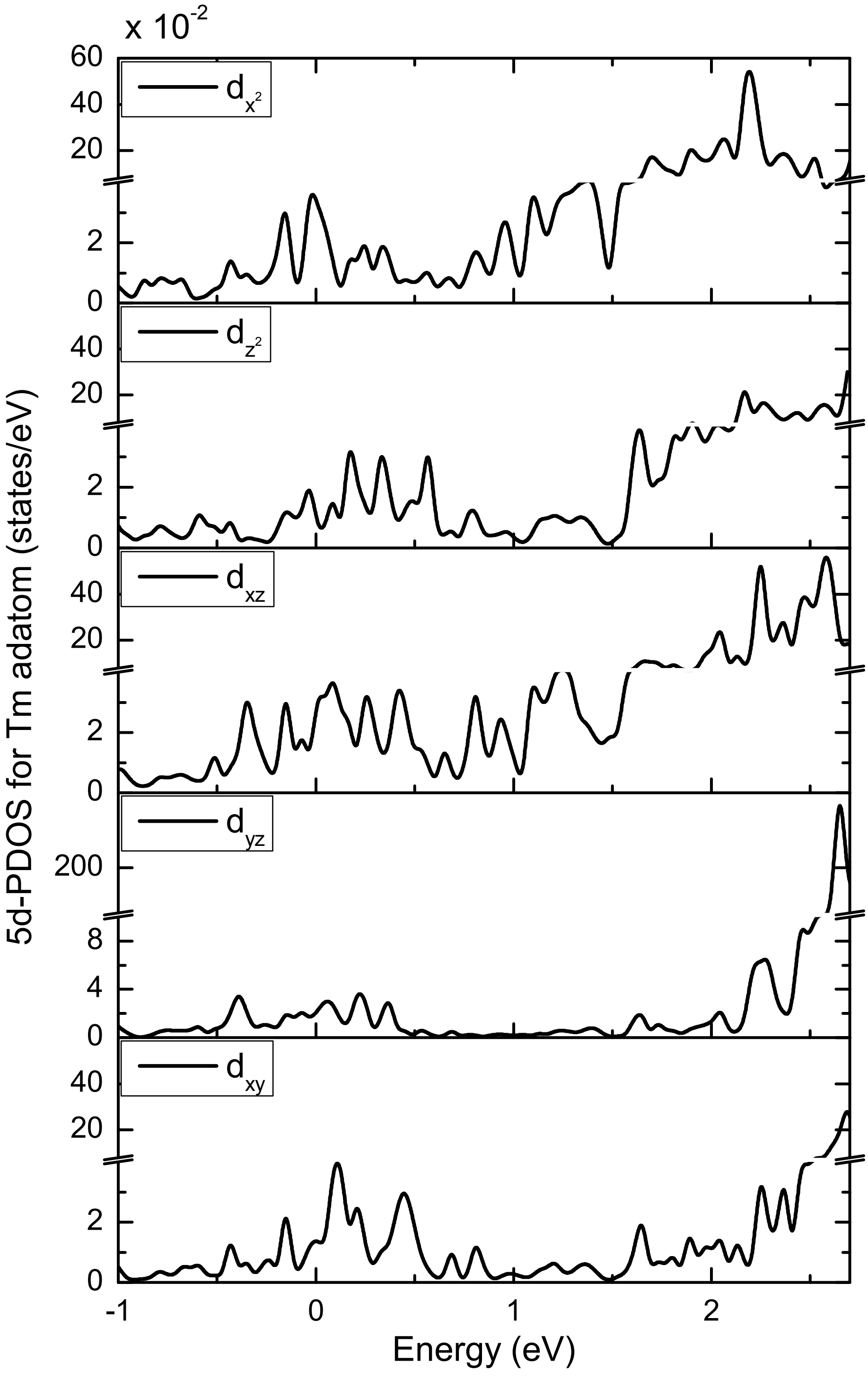

Figure S8.- The spin-averaged 5*d*-PDOS of Tm adatom at Tm/Fe-ML/(110)W.

References

1. Wortmann, D., Heinze, S., Kurz, P., Bihlmayer, G. & Blügel, S. Resolving Complex Atomic-Scale Spin Structures by Spin-Polarized Scanning Tunneling Microscopy. *Phys. Rev. Lett.* **86,** 4132–4135 (2001).

2. Kresse, G. & Furthmüller, J. Efficiency of ab-initio total energy calculations for metals and semiconductors using a plane-wave basis set. *Comput. Mater. Sci.* **6,** 15–50 (1996).

3. Perdew, J. & Zunger, A. Self-interaction correction to density-functional approximations for many-electron systems. *Phys. Rev. B* **23,** (1981).

4. Hellmann, H. A New Approximation Method in the Problem of Many Electrons. *J. Chem. Phys.* **3,** 61 (1935).

5. Blöchl, P. Projector augmented-wave method. *Phys. Rev. B* **50,** 17953–17979 (1994).

6. Liechtenstein, A. I., Anisimov, V. I. & Zaanen, J. Density-functional theory and strong interactions: Orbital ordering in Mott-Hubbard insulators. *Phys. Rev. B* **52,** R5467–R5470 (1995).

7. Hobbs, D., Kresse, G. & Hafner, J. Fully unconstrained noncollinear magnetism within the projector augmented-wave method. *Phys. Rev. B* **62,** 11556–11570 (2000).

8. Hong, S., Freeman, A. & Fu, C. Structural, electronic, and magnetic properties of clean and Ag-covered Fe monolayers on W (110). *Phys. Rev. B* **38,** 156–163 (1988).

9. Batirev, I. *et al.* Surface atomic forces and multilayer relaxation of W(001), W(110) and Fe/W(110). *Surf. Sci.* **417,** 151–158 (1998).

10. Qian, X. & Hübner, W. First-principles calculation of structural and magnetic properties for Fe monolayers and bilayers on W(110). *Phys. Rev. B* **60,** 16192–16197 (1999).

11. Costa, A., Muniz, R., Cao, J., Wu, R. & Mills, D. Magnetism of an Fe monolayer on W(110). *Phys. Rev. B* **78,** 054439 (2008).

12. Makov, G. & Payne, M. Periodic boundary conditions in ab initio calculations. *Phys. Rev. B* **51,** (1995).

13. Neugebauer, J. & Scheffler, M. Adsorbate-substrate and adsorbate-adsorbate interactions of Na and K adlayers on Al (111). *Phys. Rev. B* **46,** (1992).

14. Press, W. H., Flannery, B. P., Teukolsky, S. A. & Vetterling, W. T. *Numerical recipes. The art of scientific computing. Cambridge Univ. Press* 818 (1986). at <http://adsabs.harvard.edu/abs/1986nras.book.....P>

15. Pulay, P. Convergence acceleration of iterative sequences. The case of SCF iteration. *Chem. Phys. Lett.* **43,** 293 (1980).

16. Campbell, I. A. Indirect exchange for rare earths in metals. *J. Phys. F Met. Phys.* **2,** L47–L50 (1972).

17. Anisimov, V. I. *et al.* Coulomb repulsion and correlation strength in LaFeAsO from density functional and dynamical mean-field theories. *J. Phys. Condens. Matter* **21,** 075602 (2009).

18. Burzo, E., Chioncel, L., Tetean, R. & Isnard, O. On the R 5d band polarization in rare-earth-transition metal compounds. *J. Phys. Condens. Matter* **23,** 026001 (2011).
